# Emergency Department Patients’ COVID-19 Vaccination Status and Self-Reported Barriers

**DOI:** 10.5811/westjem.2022.1.54615

**Published:** 2022-03-18

**Authors:** Bethany W. Harvey, Kyle J. Kelleran, Heidi Suffoletto, Changxing Ma, Nan Nan, Michelle D. Penque, E. Brooke Lerner

**Affiliations:** * Jacobs School of Medicine and Biomedical Sciences, Department of Surgery, Buffalo, New York; † Jacobs School of Medicine and Biomedical Sciences, Department of Emergency Medicine, Buffalo, New York; ‡ Jacobs School of Medicine and Biomedical Sciences, Department of Orthopedics, Buffalo, New York; § State University of New York at Buffalo, School of Public Health and Health Professions, Department of Biostatistics, Buffalo, New York; ¶ Jacobs School of Medicine and Biomedical Sciences, Department of Pediatrics, Buffalo, New York

## Abstract

**Introduction:**

This study surveyed adult emergency department (ED) patients and the adult companions of pediatric patients to determine whether rates of coronavirus disease 2019 (COVID-19) vaccination were comparable to that of the general population in the region. This study also sought to identify self-reported barriers to vaccination and possible areas for intervention.

**Methods:**

A survey was administered to 607 adult ED patients or the adult companions of pediatric patients from three different regional hospitals to assess their COVID-19 vaccination status, COVID-19 vaccine barriers, and demographic information.

**Results:**

Of the 2,267 adult patients/companions considered for enrollment, we approached 730 individuals about participating in the study. Of the individuals approached, 607 (41% male; mean age 47.0+17.4 years) consented to participate. A total of 403 (66.4%) participants had received at least one vaccine dose as compared to 70% of the adult population in the county where the three hospitals were located. Of those, 382 (94.8%) were fully vaccinated and among the individuals who were partially vaccinated the majority (17 of 21) had an appointment for their second dose. Of those approached, 204 (33.6%) were not vaccinated, with 66 (10.9% of the total population) expressing an interest in becoming vaccinated while the remaining 138 did not want to be vaccinated. Of those who wanted to be vaccinated 32% were waiting for more safety data, and of those who did not want to be vaccinated 26% were concerned about side effects and risks and 28% were waiting for more safety data.

**Conclusion:**

Adult ED patients and adult companions of pediatric ED patients were vaccinated at a slightly lower rate than the general population in our county. A small but significant proportion of those who were unvaccinated expressed the desire to be vaccinated, indicating that the ED may be a suitable location to introduce a COVID-19 vaccination program.

## INTRODUCTION

Emergency departments (ED) have long acted as a safety net for the medical needs of many in modern society and, as such, EDs are often used by those who are considered to be at risk or disadvantaged.^1^–^4^ Because of this, emergency physicians have a unique opportunity to discuss and/or offer preventative services while they address the emergent needs of their patients. In some institutions, this has included offering vaccinations.^5^

The coronavirus disease 2019 (COVID-19) pandemic has put a strain on the medical system, particularly in EDs and intensive care units.^6^,^7^ Vaccinating our population against COVID-19 will be a key factor in reducing the burden of this virus on society and our health systems. The distribution of the COVID-19 vaccine began in January 2021 with the goal of getting 70–90% of the United States population vaccinated.^8^ Due to many factors, vaccinating the general population has faced significant barriers.^6^,^9^–^11^ During the initial phase of offering vaccinations in the US resource allocation was easily absorbed by those actively seeking the vaccine. Unfortunately, many parts of the US have reached saturation for delivering vaccine to those who are actively seeking it and now need to shift their public health programming to try to engage individuals who are not actively seeking the vaccine or are hesitant to get vaccinated. While generally the vaccine is now available in the US to everyone who wants to be vaccinated, it is likely that more targeted efforts will be needed to reach the remaining eligible vaccine candidates.

To best identify how to support ongoing vaccination efforts, one must understand the population barriers to vaccination and the basis of vaccine hesitancy. Sparked by the Wakefield paper published in *Lancet*, which erroneously concluded that the developmental regression associated with autism spectrum disorder may be attributed to the measles, mumps, and rubella vaccine, vaccine hesitancy was brought to the forefront of popular culture in the late 1990s.^12^ Although the article was ultimately retracted, this reignited research in multiple disciplines, including behavioral psychology, bioethics, economics, and medicine, regarding vaccine hesitancy. A comprehensive review was performed in 2011 when the World Health Organization EURO Vaccine Communications Working Group presented the 3C model of vaccine hesitancy, focusing on complacency, confidence, and convenience.^13^

Complacency refers to the areas where perceived risk of the disease is low and/or vaccination is not deemed an important aspect of prevention. Confidence refers to trust in both the individual and systems providing the vaccine, as well as the safety and efficacy of the vaccine itself. Convenience, as the name suggests, refers to commonly viewed barriers to vaccination: the availability, affordability, and global accessibility of the vaccine. Later, collective responsibility and utility calculation were added to the definition to establish the 5C model of vaccine hesitancy.^14^,^15^

Adult ED patients and the adult companions of pediatric ED patients may represent a disproportionate number of unvaccinated individuals. If this is true the ED could provide a unique setting to provide vaccinations and increase local vaccination rates. In this study we sought to determine whether the ED population is a good target for vaccination efforts and whether the rates of COVID-19 vaccination among ED patients and adult companions of pediatric patients were comparable to that of the general population in the region. We also identified self-reported barriers to vaccination and possible areas for intervention.

**Population Health Research Capsule**

What do we already know about this issue?

*Efforts in the US to vaccinate against COVID-19 have effectively reached those who want the vaccine. We now need to focus on those not actively seeking vaccination.*

What was the research question?

*Should vaccination be offered in the emergency department (ED), and are the vaccination rates lower than in the region?*

What was the major finding of the study?

*Of the ED population, 10% were not vaccinated but expressed an interest in getting vaccinated.*

How does this improve population health?

*A significant proportion of those in the ED who were unvaccinated want to be vaccinated, indicating the ED may be a suitable location to offer a vaccination program.*

## METHODS

We conducted a researcher-administered survey in three EDs. This study was approved by the institutional review board at the State University of New York at Buffalo in Buffalo, NY, with each participant providing verbal consent.

### Setting

The survey was conducted at three of the 10 hospitals in Erie County, NY, that are licensed to provide emergency care. The population of Erie County is just over 950,000 people. The institutions included were two regional comprehensive hospitals, one of which is the regional trauma center with over 65,000 visits per year, and the other the regional stroke center with over 64,000 visits per year. The third hospital is the region’s only children’s hospital with 45,000 visits per year.

### Inclusion Criteria

At the two comprehensive hospitals each adult patient in the ED was considered for enrollment, regardless of chief complaint, when research staff were available to enroll. When a patient was identified we recorded triage category and chief complaint. We then approached the patient’s clinician to determine whether the patient was able to participate. Reasons not to approach a patient included being too ill to participate, not capable of providing consent, actively receiving care, non-English speaking, being subject to infectious precautions, or sleeping. At the pediatric hospital the same procedures were followed, but the targets of the survey were the adult companions of pediatric patients. If an adult patient or the adult companion of a pediatric patient was deemed capable of being approached the researcher entered the room and obtained verbal consent.

### Data Collection

Once ability to participate and consent was established, the survey was verbally administered and the answers recorded on an iPad tablet (Apple Inc., Cupertino, CA) using Research Electronic Data Capture data management platform software 10.3.3 (REDCap, Vanderbilt University, Nashville, TN). Both categorical and open-ended questions were included. Any question that asked the participant for a reason was read as open-ended. The research assistants would listen to the subject’s open-ended response and record the answers based upon set categories. For responses that did not fit one of the given categories the research assistant documented the response. One of the authors then reviewed these answers and classified them. These classifications were then reviewed and verified by the other authors. If a general category could not be defined the response was coded as “other” for the analysis.

### Data Analysis

Once data collection was completed it was exported from REDCap and analyzed using Excel (Microsoft Corporation, Redmond, WA), SPSS (IBM Corp., Armonk, NY), and SAS 9.4 (SAS Institute Inc, Cary, NC). We used descriptive statistics, chi-square test (Fisher’s exact test), and logistic regression model to analyze the responses. There was no consideration of power for this descriptive study; however, a goal of 200 surveys at each institution was set, and enrollment continued at each site until that goal was reached. Enrollment began May 27, 2021, and ended July 11, 2021. We compared the vaccination rates in our subjects to the county-documented vaccination rate for the population 18 years and older as of July 11, 2021.^16^

We performed Pearson’s chi-square tests (Fisher’s exact tests for small group size) to compare the differences in COVID-19 vaccine status across participants of different characteristics (race, age group, education). A logistic regression model was developed to assess the effects of race, gender, ethnicity, age group, education level, insurance status, hospital site, and flu-vaccine status on the outcome variable. We categorized the outcome variable based on participants’ COVID-19 vaccine status with participants who did not receive and did not want the COVID-19 vaccine categorized as the cohort “declining vaccine,” while participants who were fully/partially vaccinated or had not yet received the vaccine but wanted to be vaccinated categorized as the group “not declining vaccine.”

## RESULTS

**Figure 1:**
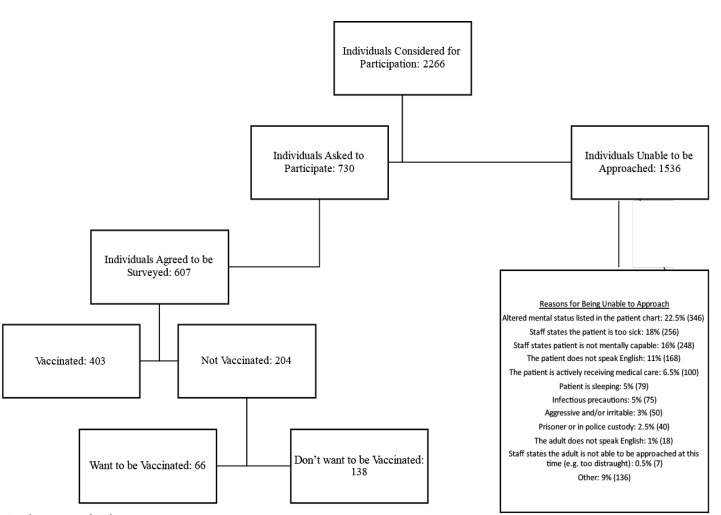
Description of study populations.

A total of 2267 adult patients/companions were considered for enrollment. We approached 730, and 607 consented to participate (Figure 1). The majority of participants were female (58%) with a mean age 47.0±17.4 years (Table 1). When compared across study sites we were not surprised to find differences in demographics since those generally aligned with the individual hospital’s catchment areas (Table 1). The adult companions of pediatric patients had the lowest vaccination rate (55%), even though the flu vaccination rates were relatively similar across all three sites. The percent of subjects who were not vaccinated but wanted to be was consistent across all sites at approximately 10%.

Of those surveyed, 403 (66.4%) had received at least one dose of vaccine, with 382 (63%) completely vaccinated. This number was slightly lower than the COVID-19 vaccination rate reported for adults in the study county, which was 70% who had received at least one dose and 65.3% who completed the series. There were 21 people who still needed a second vaccine dose to complete the series; most (80.9%) had an appointment for the second dose, while the remaining four stated they’d had side effects that kept them from getting the second shot (N = 2) or they had time or mobility issues (N = 2) that kept them from getting the second shot.

Of the 204 (33.6%) participants who were not vaccinated, 66 (10.9% of the total population surveyed) expressed interest in becoming vaccinated, while 138 (22.7% of the total population surveyed) stated they did not want to be vaccinated. The primary reasons for not getting vaccinated were that they were waiting for more safety data or they had concern with risks and side effects (Table 2). Four of the unvaccinated individuals mentioned that their reason for not getting vaccinated stemmed from a conversation with their doctor, which supported this decision. Two who wanted the vaccine but were not yet vaccinated reported that they had recently been diagnosed with COVID-19 and their doctor said to wait to get the vaccine. Two who did not want the vaccine stated they were advised against it by their physician due to medical concerns and medication issues. The chi-square test (Fisher’s exact test) results showed significant associations between age group, education level, flu-vaccine status, and COVID-19 vaccine status. Comparing those who were vaccinated to those who were not we found that those who were vaccinated tended to be older, more educated, and had previously gotten a flu vaccine (Table 3).

**Figure 2:**
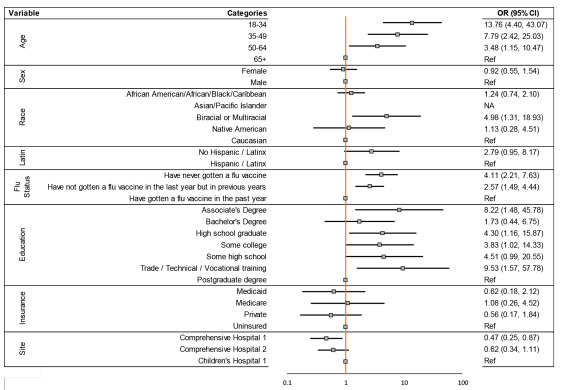
The odds ratios and 95% confidence intervals for declining COVID-19 vaccination for different demographic characteristics. No results are shown for the Asian racial group because none of the Asian participants declined the COVID-19 vaccine; thus, the odds ratio could not be estimated. *COVID-19*, coronavirus disease 2019.

The logistic regression model found that age, race, flu vaccination status, education level, and study site were all associated with declining the COVID-19 vaccine (Figure 2). Specifically, the age group 18–34 years was found to be most strongly associated with increased odds of declining the COVID-19 vaccination when compared with those over age 65 (odds ratio [OR] 13.76; 95% confidence interval [CI]: 4.40 – 43.07). Individuals who identified as biracial or multiracial had an increased odds of declining COVID-19 vaccination when compared with those who identified as White (OR 4.98; 95% CI: 1.31–18.93). Participants who had never received the flu vaccine had an increased odds of declining COVID-19 vaccination when compared to participants who had received flu vaccine the prior year (OR 4.11; 95% CI: 2.21 – 7.63). Compared with those with a postgraduate education, the odds of declining the COVID-19 vaccination were 9.53 times higher among those with trade, technical, or vocational training (95% CI: 1.57 – 57.78). Lastly, the adult patients interviewed were less likely to decline vaccination when compared to the adult companions of the pediatric patients (OR 0.47; 95% CI: 0.25 – 0.87).

## DISCUSSION

This study found that while a majority of adult ED patients and the adult companions of pediatric patients have been vaccinated for COVID-19 there was a small but not insignificant proportion of the ED population that wanted to be vaccinated but had not yet been vaccinated. Just over 10% of those surveyed expressed interest in getting vaccinated. This could be enough to consider offering the vaccine in the ED. Previous research has found that convenience plays a large role in human behavior and compliance^17^–^20^; so. it seems possible that such a program might be successful. In June 2021, the American College of Emergency Physicians developed and published toolkits for patient education^21^ and for ED implementation of vaccination programs.^22^ While EDs have given tetanus vaccines in great numbers over a long period of time,^23^ prior studies have also shown the efficacy of offering the influenza vaccine in both general and pediatric EDs. 5,24

Our finding that 37% of the ED population was not fully vaccinated aligns with two earlier studies that found 39% and 32% of ED patient population were vaccine-hesitant when asked if they would receive the vaccine.^25^,^26^ These prior studies ended in March and May 2021, respectively, while ours began in July 2021. This means that the efforts to increase comfort with vaccination and the ready availability of the COVID-19 vaccine in the intervening time may not have been impactful in this population. We identified that many of those who were not vaccinated might otherwise have done so because their concerns about side effects and safety could be addressed in the ED setting. Having an opportunity to discuss these concerns with emergency clinicians might play a role in increasing vaccination rates. Encouraging vaccination through appropriate medical counseling may impact barriers associated with confidence, complacency, risk calculation, and collective responsibility.^17^–^20^

When comparing vaccination rates between the three hospital sites, the adult companions at the pediatric hospital had the lowest vaccination rates, which was found to be significant in our multivariable model. Among those who were not vaccinated, the adult companions also had the highest rate of reporting they would not want the vaccine. One possible conclusion is that this population was younger and likely in better health than those presenting to the ED themselves for care and, therefore, may have been more likely to believe that they did not need the vaccine. The fact that many of our participants were parents of young children who were still unable to receive the vaccine enhances the importance of vaccination in this group and may represent a key opportunity for education and intervention.

It is interesting that the majority of individuals who were vaccinated had received the full series or had an appointment to complete the series. One concern with offering vaccination in the ED is that people might not obtain the second shot. Offering a single-shot vaccine in the ED is likely the most viable option; if this is not possible a system for obtaining the second shot will need to be developed. It is encouraging that our data shows that individuals are likely to be compliant with obtaining the second dose. Many pharmacies offer the opportunity to receive a second dose, regardless of where individuals received their first dose. This was often not the case when the vaccine was first available; so this too may create an opportunity for education.

In our population, more people who had the COVID-19 vaccine had previously received the flu vaccine. However, we were surprised to find some discordance with flu vaccination status and the desire to receive COVID-19 vaccination. Previous studies have noted that regularly declining the influenza vaccination closely aligns with refusal of the COVID-19 vaccine.^27^ Interestingly, we found that approximately 70% of those who said they did not want the COVID-19 vaccine had received the influenza vaccine either in the prior year or within the past few years. This may signify that, in our population, those reporting that they would not want the COVID-19 vaccine may be open to further conversations regarding specifics of their vaccine hesitancy. In fact, the most frequently reported barriers to vaccination in this population were concerns regarding side effects and the need for more safety data. With this in mind, it is possible that with the recent full US Food and Drug Administration (FDA) approval of the Pfizer vaccine, many of those who stated they would not want to get the vaccine may now be more open to COVID-19 vaccination.

Self-reported barriers to vaccination and factors contributing to vaccine hesitancy in our population focused primarily on side effects/risks, desire for more data, and believing the vaccine “does not work,” or vaccine hesitancy due to lack of confidence. A smaller percentage of our population, primarily in those who had not been vaccinated but wished to receive the vaccine, reported difficulty obtaining the vaccine or vaccine hesitancy due to convenience. There were no reported barriers regarding complacency or collective responsibility, and only four participants noted utility calculation (“let others get it first”) as a determinant in deciding not to get the vaccine. As previously noted, providing single-dose vaccinations in the ED can be a viable option to target vaccine hesitancy due to convenience.

Overcoming vaccine hesitancy secondary to confidence in both the vaccine itself and the medical/scientific community is more difficult to overcome.^9^ This is complicated by variable advice given to patients by different healthcare clinicians. One possible avenue is to focus on improving the quality of information available where it is most commonly accessed. For our population the reported top four places vaccine information was obtained was television, friends and family, physicians, and social media, in that order. Unfortunately, our population mirrors a national trend of physicians being underused as a primary source of medical information. Public health experts and the medical community need to continue to speak publicly about the safety and efficacy of vaccination to reach patients through other mediums (such as television) as well as reaching out to patients, family, and friends personally. Likewise, enhanced efforts to educate physicians and non-physician healthcare personnel in evidence-based information on the vaccine may also be important, given that four of our participants stated that their personal physicians played a role in their decision not to get vaccinated. This, in conjunction with the full FDA approval of COVID-19 vaccines, will hopefully help move the vaccine hesitant to vaccine accepting.

## LIMITATIONS

This study may be limited by the high rate of exclusions resulting in our data not representing all ED patients, especially those who could not be accessed due to infectious symptoms. Many of these patients could have had COVID-19 and been less likely to be vaccinated. However, we conducted this study when transmission in our area was low, and in fact only 4.9% of patients were excluded due to infectious symptoms. Further, given that the majority of those excluded were too sick or cognitively incapable of participation, it could be argued that the patients we captured are those most likely to be capable of considering and discussing vaccination during the course of their ED care. Nonetheless, our finding that 10% of the interviewed patients were not vaccinated but wanted to be, may not directly translate to 10% of the ED population.

It is also of concern that 123 of the individuals whom we approached to participate in the study declined. Given the contentious nature of some discussions around vaccination it is possible that those who were vaccine-averse may have been less likely to agree to discuss their vaccination status for our survey. However, if a vaccine program were started in our ED it is likely that these individuals would also decline to participate. Further, we did not ask those individuals whether they would be willing to be vaccinated if it was offered in the ED. However, these findings could be supportive for programs that want to further investigate providing COVID-19 vaccination in the ED setting.

Finally, it is important to note that vaccination can be an emotional topic for many individuals. We trained our staff and wrote our questions to be as non-judgmental as possible and to encourage individuals to share their true opinions, but it is possible that some respondents did not feel comfortable providing honest opinions.

## CONCLUSION

Adult ED patients and adult companions of pediatric ED patients were vaccinated at a slightly lower rate than the general population in our county. A small but not insignificant proportion of those who had not yet been vaccinated wanted to be vaccinated, indicating that the ED may be a suitable location to offer the COVID-19 vaccine.

## Figures and Tables

**Table 1:** Description of the included subjects compared by hospital location.

	Total N = 607	Comprehensive hospital 1 N = 201	Comprehensive hospital 2 N = 200	Children’s hospital 1 N = 206
Gender				
Male	41.3% (251)	40% (80)	60.5% (121)	24% (50)
Female	58.5% (355)	60% (121)	39% (78)	76% (156)
Other	0.2% (1)	0% (0)	0.05% (1)	0% (0)
Age				
Mean years	47.0	54.8	49.0	37.6
(±) SD	17.4	18.1	18.1	10.3
Race				
African-American/African/Black/Caribbean	28.5% (173)	33% (67)	26% (52)	26% (54)
Asian/Pacific Islander	2.5% (16)	1.5% (3)	0.5% (1)	6% (12)
Caucasian/White	60% (365)	58% (117)	62% (124)	60% (124)
Native American	2% (13)	1.5% (3)	2% (4)	3% (6)
Other	4% (22)	3% (6)	5.5% (11)	2% (5)
Biracial or Multiracial	2% (11)	1% (2)	3% (6)	1% (3)
Prefer not to answer	1% (7)	1.5% (3)	1% (2)	1% (2)
Hispanic/Latinx				
Yes	8.7% (53)	8% (17)	7% (14)	10.7% (22)
No	91.1% (553)	92% (184)	93% (186)	88.3% (183)
Prefer not to answer	0.2% (1)	0% (0)	0% (0)	0.5% (1)
Vaccination status				
Fully vaccinated	63% (382)	68% (137)	65% (130)	55% (115)
Partially vaccinated	3% (21)	5% (10)	2.5% (5)	3% (6)
Not vaccinated - want vaccine	11% (66)	13% (26)	10% (20)	10% (20)
Not vaccinated - don’t want vaccine	23% (138)	14% (28)	22.5% (45)	31% (65)
Vaccine brand				
Pfizer	30% (181)	33% (66)	31.5% (63)	25% (52)
Moderna	25% (153)	30% (61)	26% (52)	19.5% (40)
Johnson & Johnson	11% (64)	9.5% (19)	8.5% (17)	13.5% (28)
Couldn’t remember	1% (5)	0.5% (1)	1.5% (3)	0.5% (1)
Not vaccinated	34% (204)	27% (54)	32.5% (65)	41.5% (85)
Location received				
State- or county- run clinic	24% (144)	23% (47)	21.5% (43)	26% (54)
Pharmacy	19.5% (119)	24% (48)	21.5% (43)	14% (28)
Healthcare organization clinic	15% (91)	16% (32)	17% (34)	12% (25)
Physician’s office	3.5% (21)	7% (14)	1.5% (3)	2% (4)
Other	4.5% (28)	3% (6)	6% (12)	5% (10)
Not vaccinated	33.5% (204)	27% (54)	32.5% (65)	41% (85)
Internet at home				
Yes	92% (558)	90% (181)	87.5% (175)	98% (202)
No	8% (49)	10% (20)	12.5% (25)	2% (4)
Flu vaccine status				
Have gotten a flu vaccine in the past year	50.5% (307)	53% (107)	48% (96)	50.5% (104)
Have not gotten a flu vaccine in the last year but have in the past	32.5% (196)	33% (67)	31.5% (63)	32% (66)
Have never gotten a flu vaccine	17% (104)	13% (27)	20.5% (41)	17.5% (36)
Education level				
Some high school	7% (44)	10.5% (21)	6.5% (13)	5% (10)
High school graduate	36.5% (221)	33% (66)	46.5% (93)	30% (62)
Some college	23% (137)	22% (44)	19.5% (39)	26% (54)
Associate’s degree	2% (13)	3.5% (7)	0.5% (1)	2% (5)
Bachelor’s degree	20% (123)	20% (40)	19.5% (39)	21% (44)
Postgraduate degree	8% (49)	7.5% (15)	4.5% (9)	12% (25)
Technical/trade/vocational training	2.5% (15)	3.5% (7)	2% (4)	2% (4)
Other	1% (5)	0.5% (1)	1% (2)	1% (2)
Insurance type				
Private	51.5% (313)	45% (90)	50% (100)	60% (123)
Medicare	18.5% (113)	27% (55)	24% (48)	5% (10)
Medicaid	26% (156)	25% (50)	22.5% (45)	30% (61)
Uninsured	3% (18)	2% (4)	3% (6)	4% (8)
Other	1% (7)	1% (2)	0.5% (1)	2% (4)
Sources of information (Multiple selections allowed; percent based on total respondents)				
Friend/family	18% (243)	20% (92)	18% (87)	15% (64)
Social media	13% (182)	10% (47)	12% (60)	18% (75)
Primary care doctor/clinician	13% (185)	18% (82)	13% (65)	9% (38)
Newspaper	7% (97)	5% (23)	7% (34)	10% (40)
TV	21% (288)	21% (96)	24% (117)	18% (75)
Radio	4% (56)	4% (19)	5% (23)	3% (14)
Personal research	12% (163)	16% (73)	11% (56)	8% (34)
Workplace	6% (82)	3% (14)	4% (19)	12% (49)
Religious leaders	1% (14)	1% (5)	1% (6)	1% (3)
Other	4% (53)	2% (9)	4% (22)	5% (22)

*SD*, standard deviation.

**Table 2:** Self-reported barriers to COVID-19 vaccination in unvaccinated by desire to obtain vaccine.

Barrier to vaccination	Wish to get the vaccine (N = 66)	Don’t wish to get the vaccine (N = 138)
Already had COVID-19	5% (3)	4% (6)
Can’t get an appointment	6% (4)	-
Can’t get it at my desired location	2% (1)	-
Can’t get to the vaccination site	5% (3)	-
Don’t think it works	-	18% (25)
“Let others get it first”	-	3% (4)
Opposed to vaccines/medical care	-	4% (5)
Pregnancy/breastfeeding	5% (3)	3% (4)
Scheduled	3% (2)	-
Side effects/risks	18% (12)	26% (36)
Time	11% (7)	-
Waiting for more safety data	32% (21)	28% (38)
Other	15% (10)	14% (20)

*COVID-19*, coronavirus disease 2019.

**Table 3:** COVID-19 vaccination status compared to history of influenza vaccination status, education, age, and sources of Information.

	Received COVID-19 vaccination			

	Yes (N = 403)	No, but wants vaccine (N = 66)	No, don’t want vaccine (N = 138)	*P*-value (chi-square test)
Flu vaccination				
Received the flu vaccine in the past year	79.48% (244)	9.12% (28)	11.40% (35)	<0.001
Did not get a flu vaccine in the last year but has in prior years	57.14% (112)	11.73% (23)	31.12% (61)	
Never received a flu vaccine	45.19% (47)	14.42% (15)	40.38% (42)	
Education level				
Some high school	63.64% (28)	9.09% (4)	27.27% (12)	<0.001*
High school graduate	57.92% (128)	14.93% (33)	27.15% (60)	
Some college	59.85% (82)	13.87% (19)	26.28% (36)	
Associate’s degree	53.85% (7)	7.69% (1)	38.46% (5)	
Bachelor’s degree	82.93% (102)	4.88% (6)	12.20% (15)	
Postgraduate degree	91.84% (45)	2.04% (1)	6.12% (3)	
Technical/trade/vocational training	53.33% (8)	13.33% (2)	33.33% (5)	
Other	60.00% (3)	0% (0)	40.00% (2)	
Age by category				
18–34	41.52% (71)	19.30% (33)	39.18% (67)	<0.001
35–49	65.34% (115)	8.52% (15)	26.14% (46)	
50–64	80.28% (114)	6.34% (9)	13.38% (19)	
65+	87.29% (103)	7.63% (9)	5.08% (6)	
Sources of information (Multiple selections allowed; percent based on total responses)				
Friend/family	64.61% (157)	9.05% (22)	26.34% (64)	ND
Social media	54.95% (100)	12.64% (23)	32.42% (59)	
Primary care doctor/clinician	73.51% (136)	10.81% (20)	15.68% (29)	
Newspaper	61.86% (60)	6.19% (6)	31.96% (31)	
TV	62.50% (180)	10.76% (31)	26.74% (77)	
Radio	62.50% (35)	12.50% (7)	25.00% (14)	
Personal research	67.48% (110)	12.27% (20)	20.25% (33)	
Workplace	69.51% (57)	6.10% (5)	24.39% (20)	
Religious leaders	42.86% (6)	21.43% (3)	35.71% (5)	
Other	64.15% (34)	16.98% (9)	18.87% (10)	

* indicates *P*-value obtained from Fisher’s exact test.

ND indicates not calculated due to ability to choose more than one answer.
